# Food Tolerance and Quality of Eating After Bariatric Surgery—An Observational Study of a German Obesity Center

**DOI:** 10.3390/jcm14144961

**Published:** 2025-07-13

**Authors:** Alexandra Jungert, Alida Finze, Alexander Betzler, Christoph Reißfelder, Susanne Blank, Mirko Otto, Georgi Vassilev, Johanna Betzler

**Affiliations:** 1 Interdisciplinary Research Center for Biosystems, Land Use and Nutrition, Justus Liebig University Giessen, 35392 Giessen, Germany; 2 Department of Surgery, University Medical Center Mannheim, Medical Faculty Mannheim, Heidelberg University, 68167 Mannheim, Germany; 3 Department of Surgery, Bamberg Hospital, Sozialstiftung Bamberg, 96049 Bamberg, Germany; 4 German Cancer Research Center, DKFZ, 69120 Heidelberg, Germany

**Keywords:** food tolerance, bioelectrical impedance analysis, quality of life, bariatric surgery, sleeve gastrectomy, Roux-en-Y gastric bypass

## Abstract

**Background**: Bariatric surgeries, specifically laparoscopic sleeve gastrectomy (SG) and Roux-en-Y gastric bypass (RYGB), are a common intervention for morbid obesity, significantly affecting food tolerance and quality of eating. Understanding these changes is crucial for improving postoperative care and long-term success. **Methods**: This observational study at University Hospital Mannheim involved 91 patients, aged between 18 and 65 year, who underwent SG or RYGB between 2009 and 2019. Food tolerance was assessed between 25 days and 117 months after surgery using the validated score by Suter et al. (Food Tolerance Score, FTS) and an additional score evaluating tolerance to specific food groups and quality of life. Data on body composition were collected through Bioelectrical Impedance Analysis (BIA) at follow-up visits. Statistical analyses included linear mixed models to analyze the association of food tolerance with body composition changes. **Results**: The FTS indicated moderate or poor food tolerance in 62.6% of patients, with no significant differences between SG and RYGB. Considering the results of the additional score, food groups such as red meat, wheat products, raw vegetables, carbon dioxide, fatty foods, convenience food, and sweets were the most poorly tolerated food groups. A total of 57 of the participants had a baseline and follow-up BIA measurement. Postoperatively, a significant reduction in body weight and BMI as well as in BIA parameters (fat mass, lean mass, body cell mass, and phase angle) was found. Quality of life improved after bariatric surgery and 76.9% rated their nutritional status as good or excellent, despite possible food intolerances. **Conclusions**: Bariatric surgery significantly reduces weight and alters food tolerance. Despite moderate or poor food tolerance, patients reported high satisfaction with their nutritional status and quality of life. Detailed food tolerance assessments and personalized dietary follow-ups are essential for the early detection and management of postoperative malnutrition, ensuring sustained weight loss and improved health outcomes.

## 1. Introduction

Bariatric surgery is a widely established treatment option for morbid obesity and its associated comorbidities [1]. These surgical procedures work by altering the anatomy of the gastrointestinal tract, thereby restricting food intake and inducing hormonal and metabolic changes that lead to malabsorption and appetite regulation. As a result, most patients experience substantial and sustained weight loss as well as improvements in obesity-related health conditions. However, bariatric surgery can cause changes in food tolerance and quality of eating as well as eating behavior, which can potentially affect body composition and long-term health outcomes [2,3,4].

Food tolerance refers to the ability to ingest and digest various foods without adverse symptoms such as nausea, vomiting, bloating, or diarrhea. After bariatric surgery, food tolerance can be affected by the reduction in stomach size and bypassing of absorptive surfaces. Poor food tolerance may result in reduced dietary variety, impaired nutrient absorption, and non-compliance with dietary recommendations, thereby increasing the risk of weight regain or lean mass loss [3,5].

One critical concern following bariatric surgery is the development of sarcopenic obesity—a condition characterized by reduced skeletal muscle mass and function in the context of residual or recurrent adiposity. Altered food intake patterns and intolerance to protein-rich foods, along with suboptimal physical activity levels, may contribute to the progressive loss of fat-free mass. Since lean mass, particularly skeletal muscle, plays a crucial role in metabolic health, immune function, and physical independence, its preservation is essential for sustaining the benefits of bariatric surgery [6,7,8,9].

Body composition analysis—particularly through methods such as bioelectrical impedance analysis (BIA)—allows for the assessment of fat mass, lean mass, and hydration status, and is increasingly used in clinical follow-ups after bariatric procedures [6,10].

Quality of eating reflects the nutritional value of consumed foods. Post-bariatric surgery, patients are advised to follow a high-protein, low-fat, low-carbohydrate diet, rich in micronutrients. Poor dietary choices, such as high intake of processed foods and low intake of fruits and vegetables, may lead to micronutrient deficiencies and related complications [11]. Given these factors, there is increasing recognition of the need to evaluate not only weight loss outcomes, but also food tolerance and changes in body composition as part of comprehensive postoperative monitoring [7,11,12]. Standardized tools such as the Food Tolerance Score (FTS) by Suter et al. [13] offer a practical means of assessing patients’ adaptation to dietary changes. Combining such measures with BIA-based body composition tracking may help identify individuals at risk of suboptimal recovery and inform personalized nutritional interventions.

The aim of this study is to analyze the changes in quality of eating/food tolerance and body composition in obese patients after laparoscopic sleeve gastrectomy (SG) or Roux-en-Y gastric bypass (RYGB) and to evaluate the correlation between these parameters.

We hypothesize that reduced food tolerance—especially to protein-rich or fibrous foods—is associated with unfavorable changes in body composition, particularly decreased lean mass. We further expect that patients with higher food tolerance and better dietary quality will maintain a healthier body composition profile postoperatively.

## 2. Materials and Methods

### 2.1. Surgical Procedures and Postoperative Dietary Management

Sleeve gastrectomy (SG) and Roux-en-Y gastric bypass (RYGB) were performed laparoscopically and according to national guidelines and clinical standards.

Sleeve gastrectomy (SG) was selected as the surgical procedure for patients with a body mass index (BMI) greater than 50 kg/m^2^, multiple comorbidities, the need for ongoing medication, abdominal adhesions due to previous surgeries, chronic inflammatory bowel disease, or based on patient preference. Roux-en-Y gastric bypass (RYGB) was chosen for patients with a BMI below 50 kg/m^2^, gastroesophageal reflux disease (GERD), non-alcoholic steatohepatitis (NASH), or type 2 diabetes mellitus. RYGB was performed using a gastrojejunostomy and Roux-en-Y configuration (jejunojejunostomy), with an alimentary limb length of 150 cm and a biliary limb length of 60 cm.

According to clinical standards the postoperative diet is introduced in stages, beginning with a two-week liquid phase, followed by two weeks of pureed foods. Solid foods are reintroduced four weeks after surgery. Patients are advised to consume small, frequent meals and to prioritize high-protein, low-fat, and low-sugar foods. Adequate hydration is essential; however, fluids are to be consumed separately from meals to prevent early satiety and, in Roux-en-Y gastric bypass (RYGB) patients, to reduce the risk of dumping syndrome.

Proton pump inhibitors are recommended for three months postoperatively to prevent early anastomotic ulcers. Lifelong supplementation with vitamins and minerals—such as vitamin B12, iron, calcium, and vitamin D—is generally advised to prevent deficiencies resulting from altered gastrointestinal absorption. For patients experiencing difficulty maintaining weight or adapting to their diet due to food intolerances, dietary counseling is available at University Hospital Mannheim.

### 2.2. Patient Selection and Retrospective Data Analysis

The study was conducted at the Department of Surgery, University Hospital Mannheim. Ethical approval was obtained in June 2019 from the Ethics Committee II of Ruprecht-Karls-University Heidelberg (Medical Faculty Mannheim).

Eligible participants were patients who had undergone bariatric surgery—either laparoscopic sleeve gastrectomy (SG) or Roux-en-Y gastric bypass (RYGB)—at least two weeks prior to the study. Exclusion criteria included undergoing a bariatric procedure other than SG or RYGB, pregnancy or breastfeeding during the observation period, and age below 18 or above 65 years. This observational study included both retrospective and prospective data. Retrospective data—specifically bioelectrical impedance analysis (BIA) and anthropometric measurements—were collected during routine follow-up visits, which were scheduled at two weeks, and at three, six, nine, and twelve months postoperatively. BIA is a non-invasive method used to assess body composition, including fat mass, muscle mass, and total body water. It works by transmitting a low-level electrical current through the body and measuring resistance and reactance. BIA measurements were performed using a Nutriguard M device (Data Input GmbH, Pöcking, Germany). The formula applied by the manufacturer is provided in Table 1. If no BIA measurement was available, changes in anthropometric parameters (body weight and body mass index (BMI)) were analyzed. Body weight was measured using the MTS body scale (Kern & Sohn GmbH, Balingen, Germany).

### 2.3. Food Tolerance Questionnaires and Scores

Prospective data were collected through a cross-sectional study between July and December 2019, recruiting postoperative bariatric patients at routine follow-up appointments. To assess food tolerance after bariatric surgery, the validated questionnaire developed by Suter et al. in 2007 [13] was administered. This instrument evaluates overall satisfaction with dietary quality, meal timing throughout the day, tolerance to various types of food, and the frequency of vomiting or regurgitation. Based on this questionnaire, the “Suter Score” or “Food Tolerance Score” (FTS) can be calculated, enabling standardized comparisons between patients. The FTS ranges between 1 and 27, with lower scores indicating poorer food tolerance and a score of 27 reflecting no intolerance to any food type.

An additional unvalidated questionnaire was developed specifically for this study by the Interdisciplinary Research Center for Biosystems, Land Use and Nutrition at the Justus-Liebig-University Giessen, Germany. This questionnaire aimed to further evaluate food tolerance and distinguish between patients who already had low food tolerance prior to surgery and those who developed it postoperatively (see Appendix B). It assessed tolerance to 17 specific food categories, including red and white meat, fish, seafood, legumes, fruit, heated vegetables, raw vegetables/salads, rice, wheat products, dairy, nuts, fried or greasy foods, spicy foods, fast food, sweets, and carbonated beverages. Participants who reported that they could only consume a particular food with restrictions or not at all since surgery received 1 point for that item; those reporting no difficulty received 0 points. Thus, a total score of 0 indicated no newly developed food intolerances after surgery, while a score of 17 indicated intolerance to all food categories assessed. The additional questionnaire also included items on smoking and alcohol consumption, changes in physical activity, the presence and progression of comorbidities following surgery, and overall quality of life.

All questionnaires were administered once during the postoperative period.

### 2.4. Statistical Analysis

Statistical analyses were performed using the RStudio with R version 3.6.3 (29 February 2020) employing the lme4 package (version 1.1-23) and the multcomp package (version 1.4-13). Descriptive data are presented as medians with interquartile ranges (IQRs). For all statistical tests a *p*-value of <0.05 was considered statistically significant. Spearman correlation analysis was conducted to evaluate the relationship between the two food tolerance scores and to assess correlations between each score and absolute changes in body weight, BMI, lean mass, and fat mass, comparing preoperative and postoperative measurements. To investigate the association between food intolerances and changes in body composition following bariatric surgery, linear mixed-effects models were applied. Separate models were developed for anthropometric data (body weight and BMI) and body composition parameters (phase angle, lean mass, fat mass in kilograms, and body fat percentage). Fixed effects included in the models were as follows: Suter Score or additional food tolerance score (in points), gender (male vs. female), type of surgery (RYGB vs. SG), age at the time of surgery (in years), multimorbidity status (yes vs. no), and time elapsed since surgery (in months) at the time of each measurement. A random intercept for each subject was included to account for repeated measures. Results are presented as coefficient estimates with corresponding 95% confidence intervals. *p*-values were adjusted for simultaneous inference using methods for general linear hypotheses [14]. Diagnostics for the residuals of the fitted models indicated in part deviations from normal distribution and heavier upper tails. Therefore, analyses were repeated with logarithmically transformed data as regards the dependent variables. As these yielded comparable results, only the outcomes based on non-transformed data are reported.

## 3. Results

Initially, 148 patients who had undergone SG or RYGB at University Hospital Mannheim were screened for eligibility. The time between surgery and the food tolerance assessment ranged from 25 days to 117 months. A total of 57 patients were excluded due to one or more of the following reasons: failure to attend scheduled follow-up appointments, undergoing bariatric surgery less than two weeks prior to the food tolerance interview, pregnancy, age over 65 years, having undergone more than one bariatric procedure, or scheduling conflicts. Thus, 91 patients met the inclusion criteria, agreed to participate, and completed the food tolerance interview.

Of these 91 participants (referred to as Group A), 57 had at least two complete sets of BIA data, including at least one preoperative measurement (Group B), while 34 patients did not have sufficient BIA data for longitudinal analysis.

The main characteristics of the patient cohort are presented in Table 2.

### 3.1. Food Tolerance Score

Suter et al. categorize the FTS as follows: ≥24 points indicates good food tolerance, 20–24 points indicates moderate tolerance, and <20 points reflects poor food tolerance. Among the 91 participants, 37.36% (n = 34) had an FTS of ≥24, 37.36% (n = 34) scored between 20 and 24, and 25.27% (n = 23) scored below 20 postoperatively. Postoperative FTS values ranged from 9 to 27 points, while scores from the additional food tolerance questionnaire ranged from 0 to 13 points.

**Figure 1:**
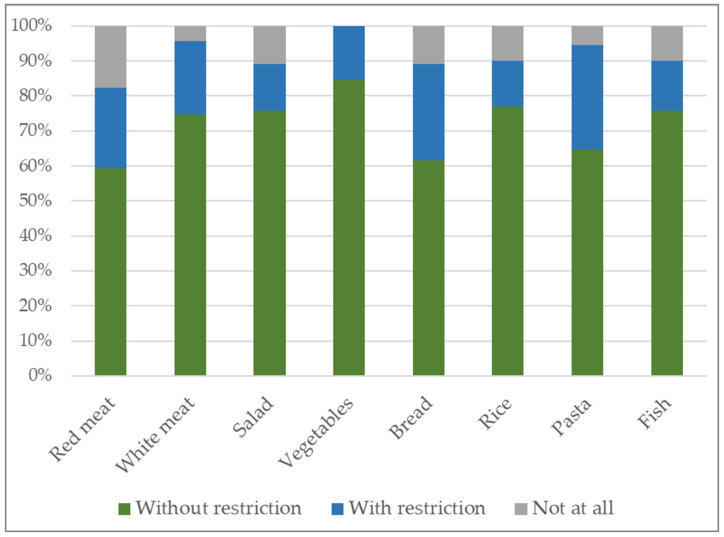
Tolerance of selected food groups after bariatric surgery (Suter Score, FTS).

In terms of satisfaction with their current nutritional status, 29.67% (n = 27) rated it as excellent, 47.25% (n = 43) as good, and 17.58% (n = 16) as sufficient. In contrast, 3.30% (n = 3) and 2.20% (n = 2) rated their postoperative nutritional status as poor or very poor, respectively. Furthermore, 51.65% (n = 47) stated that they were not able to eat all types of food. Postoperative tolerance to specific food groups—including red meat, white meat, salad, vegetables, bread, rice, pasta, and fish—is illustrated in Figure 1.

### 3.2. Additional Questionnaire

Of the 91 participants, 8.79% (n = 8) reported regular alcohol consumption, defined as drinking at least once per week. The majority (97.80%) followed an omnivorous dietary pattern, and 24.18% (n = 22) reported smoking at the time of the survey.

Postoperatively, participants engaged in a median of 120 min of physical activity per week. Specifically, 51.65% (n = 47) engaged in moderate-intensity exercise, 20.88% (n = 19) in low-intensity exercise, and 5.49% (n = 5) in high-intensity exercise. In contrast, 21.98% (n = 20) reported no physical activity at all. Compared to their preoperative levels, 86.81% (n = 79) stated that they were more physically active after surgery, while 12.09% (n = 11) reported no change. One participant noted reduced activity postoperatively due to a pre-existing knee condition unrelated to bariatric surgery. Nearly half of the study population (48.35%; n = 44) had at least two chronic comorbidities—such asatherosclerosis, arterial hypertension, diabetes mellitus type 2, dyslipidemia, gout, eating disorder, depression, asthma bronchial, reflux disease, or obstructive sleep apnea syndrome—and were therefore classified as multimorbid.

**Figure 2:**
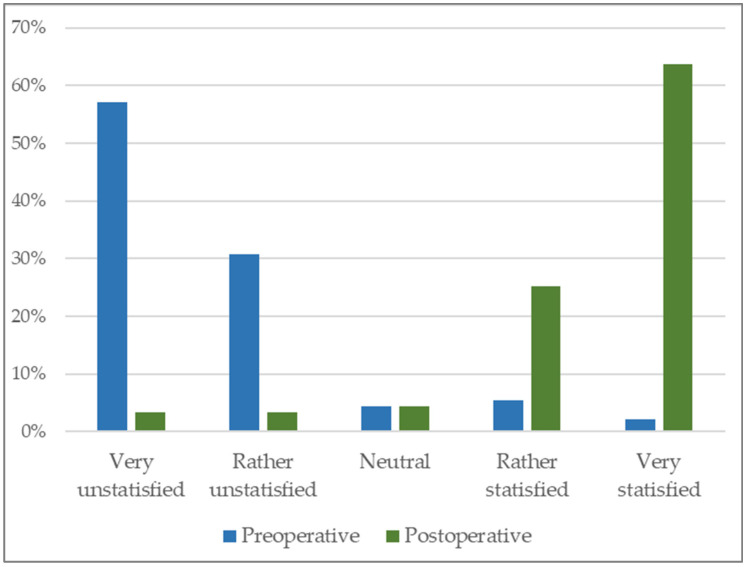
Self-assessment of quality of life (pre- and postoperatively, additional questionnaire).

Figure 2 shows participants’ self-assessed quality of life before and after bariatric surgery.

**Figure 3:**
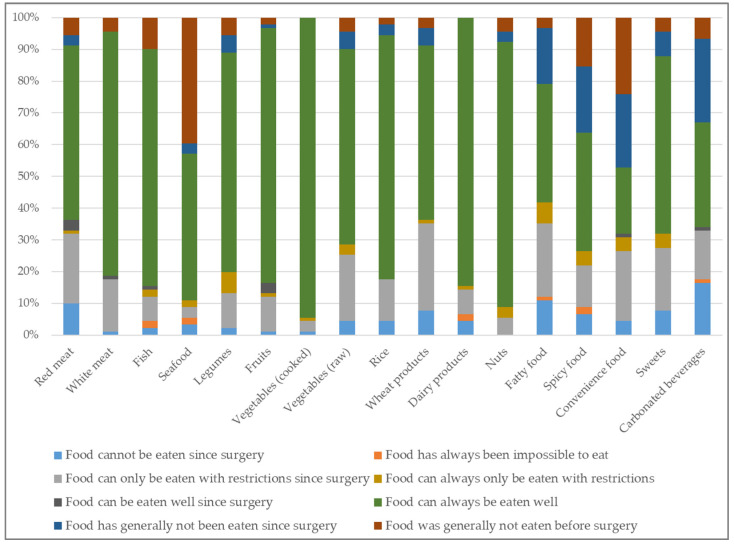
Food tolerance and its postoperative change for different food groups (additional questionnaire).

The additional questionnaire also evaluated the difference in tolerance to food categories before and after surgery (Figure 3).

To evaluate the influence of surgery on the tolerance of food groups postoperatively, a further score—referred to as the “additional score”—was calculated. This score captured all food intolerances that occurred for the first time following surgery. Among the 91 participants, 21.98% (n = 20) had a score of 0, indicating no new food intolerances postoperatively. In contrast, 48.35% (n = 44) reported between 1 and 4, 26.37% (n = 24) reported between 5 and 9, and 3.30% (n = 3) reported between 10 and 13 new food intolerances after surgery.

There was no significant difference in either the Suter Score or the additional score between the two types of bariatric surgery.

### 3.3. Spearman Correlation Analyses

The results of the Spearman correlation analyses indicated a negative correlation between the “additional score” and the difference in BMI, as well as a negative trend between the “additional score” and weight difference in Group A (Appendix A: Table A1). In Group B, correlations between the two food tolerance scores and the changes in lean mass and fat mass were examined. In this subgroup, only a trend toward a negative correlation between the FTS and lean mass difference was observed (Appendix A: Table A1). A moderate inverse correlation was found between the FTS and the additional score (Spearman’s rank correlation rho = −0.63; *p* < 0.0001). Due to this correlation, both variables were not included simultaneously in the same linear mixed-effects model.

### 3.4. Linear-Mixed Models

The results of the linear mixed-effects models are presented in Table A2, Table A3 and Table A4 in the Appendix A. The models using either the Suter Score or the additional score yielded nearly identical results for each dependent variable. Neither the Suter Score nor the additional score was significantly associated with anthropometric or body composition parameters over time after adjusting for gender, type of surgery, age at the time of surgery, multimorbidity, and time since surgery.

However, a significant reduction in body weight, BMI, and BIA parameters (fat mass, lean mass, body cell mass, and phase angle) was observed over time, even after adjusting for multiple testing. Prior to adjustment for multiple testing, SG was associated with higher body weight, BMI, and fat mass compared to Roux-en-Y gastric bypass (RYGB); however, after adjustment, only the difference in absolute fat mass remained statistically significant. Female sex was associated with lower body weight, lower absolute lean mass, lower BCM, and higher relative fat mass. After correction for multiple testing, the association with body weight was no longer statistically significant. Age at the time of surgery showed an effect on lean mass, BCM, phase angle, and ECM/BCM index, although only the latter three remained significant after adjustment. Multimorbidity showed no significant effect on anthropometric or body composition parameters.

## 4. Discussion

Food tolerance is a critical determinant of long-term success after bariatric surgery, influencing not only nutritional intake but also quality of life (QOL) and overall health outcomes. Postoperative food intolerances may hinder adequate nutrient consumption, compromise protein intake, and increase the risk of lean mass loss and micronutrient deficiencies. Therefore, evaluating food tolerance through structured tools is essential for optimizing postoperative care and nutritional counseling [13,15].

This study is among the first to demonstrate a comprehensive assessment of food tolerance using both the established Food Tolerance Score (FTS) by Suter et al. [13] and a newly developed, more comprehensive “additional score”. The additional score expands upon the FTS by including a broader range of food groups (e.g., carbonated beverages, sweets, raw vegetables), as well as behavioral and lifestyle factors such as alcohol use, smoking, physical activity, and subjective quality of life. While the Suter Score provides a well-validated framework for assessing basic tolerance and satisfaction, the additional score enhances interpretive value by differentiating between patients whose food intolerances have clinical significance (e.g., leading to protein deficiency or weight regain) and those for whom the intolerances are less impactful. Unlike the FTS, the additional score includes preoperative baseline comparison, making it possible to identify new intolerances that emerge after surgery.

The most frequently poorly tolerated food groups were red meat, wheat products, raw vegetables, and high-fat foods. These results align with previous findings and highlight that intolerances often involve nutritionally less critical or even unfavorable foods [4,16]. A negative correlation was observed between the additional score and BMI reduction over time, suggesting that higher food intolerance may initially impair weight loss. This aligns with findings from Yue et al. [17], who reported associations between lower food enjoyment or increased intolerance and slower weight loss. However, in our study, this association did not reach significance in adjusted models, potentially due to adaptive behaviors such as improved self-monitoring or avoidance of problematic foods. Notably, neither score correlated significantly with BIA-derived nutritional markers, indicating that not all reported intolerances translate into nutritional deficiencies.

The Food Tolerance Score (FTS) [13] was devised to quantify food tolerance after bariatric surgery. This scoring system offers a comprehensive assessment that considers multiple facets, including self-reported food intake quality and the ability to consume diverse food groups. Suter et al. established this score in 2007 with a study of 900 patients undergoing bariatric surgery (laparoscopic gastric banding, GB, and RYGB). They observed that food tolerance was significantly lower after GB than after RYGB, although it improved over time.

In the present study, SG and RYGB did not show significantly different results in the Suter score or the additional score. This contradicts several studies comparing bariatric surgical techniques in regard to postoperative food tolerance [18,19,20]. Pintor-de-la-Maza et al. published the results of a prospective study that showed better food tolerance after biliopancreatic diversion (BPD) in comparison to RYGB and SG [3]. In this study, 66 patients were included and BPD showed better results in the Suter score as well as BIA results after 6 and 12 months. The Suter scores of SG and RYGB did not differ significantly after 6, 12, and 24 months after surgery. The better results of BPD regarding food tolerance and postoperative vomiting might be due to the larger pouch that is built in BPD compared to RYGB. Other studies show similar results to our study regarding food tolerance after different bariatric procedures [5,21]. In a prospective, randomized-controlled trial from 2022, Medeiros et al. showed that the Suter score did not differ in elderly morbidly obese patients (>65 years) undergoing RYGB or SG [21]. They also showed that weight loss was higher after RYGB than after SG, which is also consistent with our findings.

Using the validated FTS, 37.36% of our patients exhibited moderate food tolerance (20–24 points) and 25.27% had poor tolerance (<20 points). Despite this, 76.92% rated their dietary situation as “good” or “excellent,” reflecting a discordance between objective score and subjective perception. This mismatch underscores the need for more nuanced tools that account not only for the presence of intolerance but also for its perceived impact on daily life.

Despite high numbers of intolerances, 64% of our patients also reported high QOL in the additional score, suggesting that factors beyond food tolerance (e.g., increased mobility, social integration) contribute significantly to postoperative well-being. This is consistent with Yue et al., who also reported QOL improvement despite impaired tolerance [17].

These contradicting results of food tolerance scores (FTS and additional score) and subjective perception of quality of life indicate that other questionnaires might be needed to evaluate food tolerance after bariatric surgery more accurately. New questionnaires might include questions about the impact of each respective food intolerance and symptom on QOL. With assigning different score values to the answers (e.g., duplication of the respective questions points if QOL is affected by an intolerance to a certain food group), this might help to evaluate more accurately if a food intolerance leads to problems in the daily lives of bariatric patients or not.

In 2022, Lewis et al. presented a new 27-item instrument to measure changes in enjoyment, craving, and intolerance for nine food/beverage categories after bariatric surgery (Bariatric Surgical Alterations in Tolerability, Enjoyment and Cravings in the Diet, BSATED) [15]. The BSATED questionnaire was validated in a large longitudinal survey study (Bariatric Experience Long Term, BELONG) [22,23] with 999 participants undergoing bariatric surgery. In the BELONG study, participants reported reduced enjoyment and craving for high-fat meats (62%), grains (54%), confectioneries, and other sweet treats (e.g., candy bars, chocolate, ice cream) (52%), as well as sweet baked goods (48%) twelve months post-surgery. These alterations were more prevalent in individuals undergoing RYGB in contrast to those undergoing SG. Participants who reported decreased enjoyment and craving for those foods and beverages that post-bariatric patients are counseled to reduce or avoid had greater total weight loss at 12–18 months after surgery. Less tolerability was only reported for higher-fat meats (49%) 12 months after surgery. For all other food/beverage categories, participants mostly reported no changes for tolerability. Although this instrument has some limitations (e.g., food categories familiar to people in the Southern California region of the U.S. were used, so far only use after surgery and no comparison between pre- and postoperative food consumption) it might be very interesting to use this questionnaire in a bariatric cohort and combine it with BIA measurements to evaluate if not only food tolerances but also food enjoyment and cravings might lead to altered nutritional status and body composition after bariatric surgery. The findings of this study correspond mostly to the findings of our cohort. In our study cohort, the most not tolerated food groups after bariatric surgery also included meat as well as high-fat food groups. As the BELONG study also reported less enjoyment and craving for wheat products and sweets (two of the worst tolerated food groups after bariatric surgery in our study cohort) it would have been interesting to use the BSATED questionnaire in our study cohort to evaluate if the intolerance to these food groups were only defined by lack of enjoyment or disgust.

Adequate protein intake is a key component of post-bariatric nutrition. The German S3 guideline recommends a minimum of 60 g/day following bariatric surgery [24]. Although our study did not assess protein intake directly, many food groups with high intolerance rates (e.g., wheat products, raw vegetables, sweets, carbonated beverages, fatty and convenience foods) are low in protein. Notably, reduced meat tolerance—observed in both the FTS and additional score—may compromise protein intake. While some studies report no direct link between food intolerance and inadequate protein intake [25,26], others highlight that insufficient protein consumption post-surgery can lead to fat-free mass (FFM) and muscle loss, potentially increasing morbidity [8,12,27,28]. In our cohort, a decline in lean mass over time correlated with longer postoperative intervals, likely reflecting prolonged suboptimal protein intake due to reduced total intake and altered food preferences. The ongoing PROMISE trial investigates whether a protein supplement can mitigate FFM loss after RYGB [29]. Regardless of its possibly very interesting results, our BIA data results underscore the need for continued follow-up in a nutritional outpatient or multidisciplinary bariatric setting to detect and address protein deficiency and muscle loss early.

The findings of our study should inform postoperative follow-up care. Patients with multiple new food intolerances—particularly those involving protein-rich foods like meat or legumes—should be prioritized for early dietary counseling. Registered dietitians should assess not only what foods are poorly tolerated but how these intolerances affect nutrient intake, body composition, and daily functioning.

This study is limited by its retrospective design, modest sample size, and lack of validation for the additional questionnaire. The variable time frame between surgery and the questionnaire survey especially limits the validity of the results. Nevertheless, by integrating subjective QOL, structured food tolerance assessment, and BIA data, this study provides novel insights into the multifaceted nature of postoperative dietary adaptation.

In conclusion, food intolerance is common after bariatric surgery but does not uniformly lead to reduced quality of life or impaired nutritional status. Standardized tools like the FTS, when supplemented with more comprehensive instruments, can improve the accuracy of postoperative monitoring. Our findings support the integration of food tolerance assessments into routine follow-up and highlight the importance of tailored dietary counseling to address the individual needs of bariatric patients. Further research should focus on refining these assessment tools and exploring their predictive value for long-term surgical outcomes.

## Figures and Tables

**Table 1:** Formulas for BIA parameters as specified by Data Input GmbH.

BIA Parameter	Formula
Body Water (kg)	Height^2^/(Resistance × k1)
Lean Mass (kg)	Total Body Water/0.73
Fat Mass (kg)	Body Weight—Lean Mass
Phase Angle (°)	Arctan (Reactance/Resistance) × 180/pi
Body Cell Mass (kg)	Lean Mass × Phase Angle × k2
Extracellular Mass (kg)	Lean Mass—Body Cell Mass
Cellular Component (%)	(Body Cell Mass × 100)/Lean Mass

k1 and k2 are distinct dependent correction factors or variables and are proprietary information of the company; gender and age are partially or entirely taken into account.

**Table 2:** Population characteristics.

	Group A ^1^		Group B ^2^	
Number of subjects	91		57	
RYGB	71		49	
SG	20		8	
Female	70		43	
Male	21		14	
	Preoperative	Postoperative	Preoperative	Postoperative
Age (years)	44 (37–53)	45 (38–54)	45 (37–55)	48 (39–56)
Body weight (kg)	130.2 (121.8–145.1)	100.8 (92.45–112.0)	130.3 (123.3–149.7)	102.8 (92.6–116.5)
BMI (kg/m^2^)	45.33 (42.24–49.86)	35.25 (30.72–39.75)	45.47 (42.61–50.55)	35.26 (31.51–41.05)
Lean mass (kg)	(-)	(-)	66.90 (62.08–78.60)	62.70 (57.40–68.90)
Body water (kg)	(-)	(-)	49.00 (46.00–57.60)	46.00 (42.40–50.80)
Phase angle (°)	(-)	(-)	5.40 (5.00–6.00)	4.80 (4.50–5.10)
ECM (kg)	(-)	(-)	34.0 (31.0–39.1)	34.40 (30.90–38.50)
BCM (kg)	(-)	(-)	33.30 (30.50–40.10)	28.20 (25.50–33.70)
ECM/BCM index	(-)	(-)	1.05 (0.92–1.14)	1.20 (1.11–1.28)
Fat mass (kg)	(-)	(-)	65.30 (54.60–75.90)	42.30 (27.80–52.10)
Fat mass (%)	(-)	(-)	48.10 (43.20–53.00)	40.30 (31.70–45.70)
Months after surgery at the time of food tolerance interview	9 (5–21)		12 (6–41)	
FTS	22 (20–25)		22 (20–25)	
Additional score	3 (1–5)		3 (1–5)	

^1^ Patients with interview on food tolerance and quality of life; ^2^ patients with interview on food tolerance and quality of life and at least two complete sets of BIA data; values presented as median (IQR); RYGB = Roux-en-Y gastric bypass; SG = sleeve gastrectomy; ECM = extracellular cell mass; BCM = body cell mass; FTS = Food Tolerance Score (Suter Score).

## Data Availability

The data presented in this study are available on request from the corresponding author due to data privacy reasons.

## Appendix A

**Table A1:** Correlation of food tolerance scores with anthropometric and body composition parameters.

	Suter Score (FTS)		Additional Score	
	Spearman Correlation Coefficient	*p*-Value	Spearman Correlation Coefficient	*p*-Value
Age at the time of surgery (years)	0.25	0.016	−0.22	0.039
Weight difference (kg)	0.03	0.468	−0.20	0.057
BMI difference (kg/m^2^)	0.08	0.744	−0.23	0.028
Lean mass difference (kg)	−0.23	0.084	0.11	0.415
Fat mass difference (kg)	0.06	0.654	−0.21	0.117

Differences in anthropometric (group A—91 subjects) and body composition (group B—57 subjects) parameters were calculated from preoperative to the last postoperative measurements.

**Table A2:** Results of the linear mixed-effects models for long-term determinants of anthropometric data.

	Body Weight (kg) (*n* = 91; 389 Records)		BMI (kg/m^2^) (*n* = 91; 389 Records)	
	CE	95% CI	CE	95% CI
Intercept	146.79 ***	[115.09, 178.51]	44.57 ***	[34.06, 55.09]
Score by Suter et al. (points)	−0.26	[−1.46, 0.94]	−0.15	[−0.55, 0.24]
Gender (male vs. female)	−11.57	[−21.9, −1.24]	1.43	[−1.99, 4.86]
Type of surgery (RYGB vs. SG)	13.35 †	[3.05, 23.63]	4.04	[0.63, 7.46]
Age at the time of surgery (years)	−0.36	[−0.81, 0.09]	−0.06	[−0.21, 0.09]
Multimorbidity (no vs. yes)	>−0.01	[−9.10, 9.12]	1.06	[−1.96, 4.08]
Time since surgery (months)	−0.38 ***	[−0.48, −0.28]	−0.13 ***	[−0.16, −0.09]
				
Intercept	142.50 ***	[120.83, 164.16]	41.97 ***	[34.78, 49.16]
Additional score (points)	−0.24	[−1.78, 1.30]	−0.12	[−0.63, 0.39]
Gender (male vs. female)	−10.36	[−20.63, −0.08]	2.10	[−1.31, 5.52]
Type of surgery (RYGB vs. SG)	13.65 †	[3.34, 23.94]	4.21	[0.79, 7.63]
Age at the time of surgery (years)	−0.40	[−0.84, 0.04]	−0.08	[−0.23, 0.06]
Multimorbidity (no vs. yes)	0.30	[−8.70, 9.32]	1.23	[−1.76, 4.23]
Time since surgery (months)	−0.38 ***	[−0.48, −0.28]	−0.13 ***	[−0.16, −0.09]

Data are presented as coefficient estimates (CEs) and 95% confidence intervals (CIs, 95%) and reported *p*-values were adjusted for simultaneous inference. † *p* < 0.10 (indicating trend); *** *p* < 0.001.

**Table A3:** Results of the linear mixed-effects models for long-term determinants of BIA data (lean mass and fat mass).

	Lean Mass (kg) (*n* = 57; 289 Records)		Fat Mass (kg) (*n* = 57; 289 Records)		Fat Mass (%) (*n* = 57; 289 Records)	
	CE	95% CI	CE	95% CI	CE	95% CI
Intercept	89.39 ***	[71.56, 107.22]	53.95 *	[18.23, 89.9]	36.22 ***	[20.28, 52.29]
Score by Suter et al. (points)	0.17	[−0.47, 0.81]	0.02	[−1.29, 1.31]	−0.02	[−0.60, 0.56]
Gender (male vs. female)	−20.20 ***	[−25.44, −14.95]	8.80	[−1.77, 19.34]	10.96 ***	[6.23, 15.66]
Type of surgery (RYGB vs. SG)	3.16	[−3.50, 9.80]	20.89 * ‖	[7.43, 34.25]	6.01	[<0.01, 11.98]
Age at the time of surgery (years)	−0.23	[−0.45, −0.01]	−0.25	[−0.69, 0.19]	−0.05	[−0.25, 0.15]
Multimorbidity (no vs. yes)	0.41	[−4.31, 5.12]	0.73	[−8.77, 10.23]	−0.44	[−4.68, 3.81]
Time since surgery (months)	−0.08 ***	[−0.11, −0.05]	−0.24 ***	[−0.32, −0.16]	−0.10 ***	[−0.14, −0.07]
						
Intercept	93.31 ***	[82.92, 103.69]	54.71 ***	[34.01, 75.43]	36.04 ***	[26.80, 45.30]
Additional score (points)	−0.06	[−0.83, 0.70]	−0.66	[−2.19, 0.87]	−0.29	[−0.97, 0.39]
Gender (male vs. female)	−20.65 ***	[−25.81, −15.48]	10.56	[0.26, 20.86]	11.81 ***	[7.20, 16.41]
Type of surgery (RYGB vs. SG)	2.81	[−3.95, 9.55]	22.78 ** ‖	[9.24, 36.22]	6.91	[0.87, 12.92]
Age at the time of surgery (years)	−0.22	[−0.43, >−0.01]	−0.25	[−0.67, 0.17]	−0.05	[−0.24, 0.14]
Multimorbidity (no vs. yes)	0.09	[−4.47, 4.65]	1.06	[−8.02, 10.16]	−0.24	[−4.30, 3.82]
Time since surgery (months)	−0.08 ***	[−0.12, −0.05]	−0.24 ***	[−0.32, −0.16]	−0.10 ***	[−0.14, −0.07]

Data are presented as coefficient estimates (CEs) and 95% confidence intervals (CIs, 95%) and reported *p*-values were adjusted for simultaneous inference. * *p* < 0.05; ** *p* < 0.01; *** *p* < 0.001. ‖ This association was not significant when the dependent variable was logarithmically transformed.

**Table A4:** Results of the linear mixed-effects models for long-term determinants of BIA data (phase angle, BCM, and ECM/BCM index).

	Phase Angle (°) (*n* = 57; 289 Records)		BCM (kg) (*n* = 57; 289 Records)		ECM/BCM Index (*n* = 57; 289 Records)	
	CE	95% CI	CE	95% CI	CE	95% CI
Intercept	7.05 ***	[5.52, 8.58]	48.34 ***	[39.71, 56.98]	0.61 *	[0.20, 1.02]
Score by Suter et al. (points)	−0.02	[−0.07, 0.04]	0.04	[−0.28, 0.35]	0.01	[−0.01, 0.02]
Gender (male vs. female)	−0.43	[−0.88, 0.02]	−11.15 ***	[−13.70, −8.60]	0.10	[−0.03, 0.22]
Type of surgery (RYGB vs. SG)	−0.31	[−0.89, 0.26]	1.05	[−2.20, 4.29]	0.07	[−0.09, 0.22]
Age at the time of surgery (years)	−0.02 †	[−0.04, −0.01]	−0.19 **	[−0.30, −0.09]	0.01	[<0.01, 0.01]
Multimorbidity (no vs. yes)	−0.14	[−0.55, 0.26]	−0.24	[−2.53, 2.07]	0.02	[−0.09, 0.13]
Time since surgery (months)	−0.01 ***	[−0.01, >−0.01]	−0.06 ***	[−0.09, −0.04]	<0.01 **	[<0.01, <0.01]
						
Intercept	6.67 ***	[5.78, 7.56]	49.15 ***	[44.12, 54.16]	0.78 ***	[0.55, 1.02]
Additional score (points)	0.02	[−0.04, 0.09]	0.07	[−0.30, 0.44]	−0.01	[−0.03, 0.01]
Gender (male vs. female)	−0.44	[−0.88, <0.01]	−11.48 ***	[−13.98, −8.98]	0.10	[−0.02, 0.22]
Type of surgery (RYGB vs. SG)	−0.34	[−0.91, 0.24]	0.73	[−2.56, 4.01]	0.08	[−0.07, 0.24]
Age at the time of surgery (years)	−0.03 *	[−0.04, −0.01]	−0.19 **	[−0.29, −0.09]	0.01 *	[<0.01, 0.01]
Multimorbidity (no vs. yes)	−0.12	[−0.51, 0.27]	−0.36	[−2.55, 1.86]	0.01	[−0.10, 0.11]
Time since surgery (months)	−0.01 ***	[−0.01, >−0.01]	−0.06 ***	[−0.09, −0.04]	<0.01 **	[<0.01, <0.01]

Data are presented as coefficient estimates (CEs) and 95% confidence intervals (CIs, 95%) and reported *p*-values were adjusted for simultaneous inference. † *p* < 0.10 (indicating trend); * *p* < 0.05; ** *p* < 0.01; *** *p* < 0.001.

## Appendix B. Additional Questionnaire (Not Validated)

Participant ID: ___________ Date: ___________ Months post-op:________________

Type of surgery: ______________

Gender: □ Female □ Male □ Diverse

1. How well do you tolerate the following foods?

Do you generally not eat these foods for taste or ethical reasons?

Do you experience restrictions such as nausea and vomiting?

Have you only experienced these restrictions since your surgery?

	I generally do not eat	I can eat well	I can eat with restrictions	I cannot eat at all	I experience restrictions since surgery
Red meat					
White meat					
Fish					
Seafood					
Legumes					
Fruits					
Vegetables (heated)					
Vegetables/Salad (raw)					
Rice					
Wheat products					
Dairy products					
Nuts					
Fried/fatty foods					
Spicy foods					
Convenience foods/fast food					
Sweets					
Carbonated drinks					

2. Are you currently following a specific diet?

□ Omnivore (“All-eater”)

□ Vegetarian (no meat, no fish, but dairy products and eggs)

□ Vegan (exclusively plant-based foods)

□ Other, namely: ____________________________________

3. How do you rate your dietary behavior since your surgery? (Multiple responses possible)

□ It has not changed; I eat just as before

□ I eat more vegetables

□ I eat more fruits

□ I eat more convenience foods/fast food

□ I eat several small meals throughout the day

□ I often have no appetite

□ I primarily consume liquid food

□ I consume more food products enriched with vitamins and minerals

□ I take more dietary supplements

4. Do you smoke?

□ Yes

□ No

□ Not anymore since (MM/YYYY) ____________

5. Do you drink alcohol regularly (at least once a week)?

□ Yes

□ No

6. How do you rate your current daily activity overall?

□ Predominantly sedentary activity

□ Sedentary, sometimes standing and walking activity

□ Predominantly standing and walking activity

□ Hard physical activity

7. How many minutes per week do you currently exercise?

_________________

8. At what intensity do you normally do your sport?

□ low

□ moderate

□ high

9. How would you rate your exercise behavior since your surgery?

□ It has not changed; I move just as much as before

□ I move more

□ I move less

10. Do you currently suffer from one or more of the following diseases?
  	Yes	No	Medicinally treated	Change after surgery
  Atherosclerosis				
  High blood pressure				
  Type 2 diabetes mellitus				
  Dyslipidemia				
  Gout				
  Eating disorder (Binge eating…)				
  Depression				
  Asthma bronchiale				
  Heartburn/Reflux				
  Sleep apnea syndrome				

11. Did you take diuretics (dehydration tablets) in the week before the body composition measurements at Mannheim University Hospital?

□ Yes, regularly

□ Yes, sometimes

□ No

12. How do you rate your quality of life before your surgery?

□ Very satisfied

□ Rather satisfied

□ Neutral

□ Rather dissatisfied

□ Very dissatisfied

Why do you think this is the case?

______________________________

13. How do you rate your quality of life since your surgery?

□ Very satisfied

□ Rather satisfied

□ Neutral

□ Rather dissatisfied

□ Very dissatisfied

Why do you think this is the case?

______________________________
